# *In vivo* Brainstem Imaging in Alzheimer’s Disease: Potential for Biomarker Development

**DOI:** 10.3389/fnagi.2018.00266

**Published:** 2018-09-11

**Authors:** David J. Braun, Linda J. Van Eldik

**Affiliations:** ^1^Sanders-Brown Center on Aging, University of Kentucky, Lexington, KY, United States; ^2^Spinal Cord and Brain Injury Research Center, University of Kentucky, Lexington, KY, United States; ^3^Department of Neuroscience, University of Kentucky, Lexington, KY, United States

**Keywords:** Alzheimer’s disease, brainstem, neuroimaging, imaging, *in vivo*, locus coeruleus, raphe nucleus, biomarker

## Abstract

The dearth of effective treatments for Alzheimer’s disease (AD) is one of the largest public health issues worldwide, costing hundreds of billions of dollars per year. From a therapeutic standpoint, research efforts to date have met with strikingly little clinical success. One major issue is that trials begin after substantial pathological change has occurred, and it is increasingly clear that the most effective treatment regimens will need to be administered earlier in the disease process. In order to identify individuals within the long preclinical phase of AD who are likely to progress to dementia, improvements are required in biomarker development. One potential area of research that might prove fruitful in this regard is the *in vivo* detection of brainstem pathology. The brainstem is known to undergo pathological changes very early and progressively in AD. With an updated and harmonized AD research framework, and emerging advances in neuroimaging technology, the potential to leverage knowledge of brainstem pathology into biomarkers for AD will be discussed.

## Introduction

Dementia is a global public health crisis, affecting around 47 million people worldwide and projected to nearly triple by 2050 as the global population increases in average age (World Health Organization, 2017). Alzheimer’s disease (AD) is the most common cause of aging-related dementia, accounting for 60–80% of cases (Alzheimer’s Association, 2017). Even a relatively small reduction or delay in AD incidence therefore has the potential to dramatically reduce dementia burden (Barnes and Yaffe, 2011). Unfortunately, current AD treatments are only moderately and transiently effective, and many promising treatments have fallen short in late-stage clinical trials (Mehta et al., 2017). One major obstacle is the need for refined early-stage biomarkers that can reliably predict who within the AD spectrum will develop dementia. The brainstem has long been recognized as a site with early and significant susceptibility to AD-type neuropathological change (Mann, 1983), but it has been difficult to access this region *in vivo*. Advances in non-invasive neuroimaging techniques are beginning to allow antemortem assessment of this crucial region, thereby opening the possibility of using early brainstem changes as AD biomarkers. This might extend the window in which preclinical AD is detectable or, in conjunction with other biomarkers, better define those patients most likely to experience cognitive dysfunction in the future. This concept will be discussed in light of the current AD research framework and recent neuroimaging advances.

## Alzheimer’s Disease Pathology and Diagnostic Criteria

The essential neuropathologic features of AD consist of extracellular deposits of amyloid beta peptide (Aβ), and intracellular accumulations of modified tau protein called neurofibrillary tangles (NFTs). These pathologies are accompanied by neuronal injury and neurodegeneration, leading to progressive cognitive impairment and culminating in dementia. An updated clinical staging of AD was defined in 2011 by a joint workgroup of the National Institute on Aging and the Alzheimer’s Association (NIA-AA) (Albert et al., 2011; McKhann et al., 2011; Sperling et al., 2011; Montine et al., 2012). Associated with this, the NIA-AA published neuropathologic guidelines to score AD-associated neuropathology as well as assess commonly appearing comorbid pathologies (Montine et al., 2012). Importantly, the 2011 guidelines also formalized the use of imaging and cerebrospinal fluid (CSF) biomarkers to define “preclinical AD,” wherein an individual has abnormal AD-type pathological changes and no or only subtle cognitive/behavioral symptoms. Validated *in vivo* markers for AD-type neuropathologic change include CSF levels of Aβ_42_ and phosphorylated tau, increased positron emission tomography (PET) ligand binding for amyloid or tau within the cortex, glucose hypometabolism as measured by fluorodeoxyglucose PET (FDG-PET), and brain atrophy as measured by structural magnetic resonance imaging (MRI) (Tan et al., 2014). This biomarker-defined preclinical AD framework is particularly useful as the preclinical phase can extend more than a decade before the onset of clinically defined symptoms (Quiroz et al., 2018), and therefore represents a promising window for therapeutic intervention.

The disparity between reference to AD as a clinical syndrome versus a neuropathologic phenomenon has led to some confusion, however. For example, around 30% of patients clinically diagnosed with AD do not have significant AD neuropathology at autopsy and a similar proportion of cognitively unimpaired patients do (Davis et al., 1999; Neuropathology Group, 2001). In an attempt to harmonize definitions and clarify research efforts, the NIA-AA recently released an updated 2018 framework, building upon the 2011 biomarker-based approach in defining preclinical AD (Jack et al., 2018; Khachaturian et al., 2018; Knopman et al., 2018; Silverberg et al., 2018). This new system categorizes all patients by the presence or absence of biomarker-based pathology in three categories to generate an “ATN” profile: A for amyloid, T for tau, and N for neurodegeneration. Additionally, there is a cognitive staging scheme of cognitively unimpaired, mild cognitive impairment (MCI), or dementia overlaid on the ATN biomarker profiles (**Table 1**). An individual must have biomarker group A pathology (reduced CSF Aβ_42_, increased amyloid PET, etc.) to be placed on the AD spectrum, irrespective of cognitive or other pathological changes. The framework is flexible in that it can incorporate new ATN biomarkers as they become validated, or entirely new biomarker categories. As will be discussed below, neuropathological data indicate that neuroimaging-accessible changes within the brainstem may be useful additions to the T or N biomarker groupings.

**Table 1:** Overview of NIA-AA ATN biomarker scores and cognitive staging.

Cognitive stage	A	T	N	Description of ATN profile and cognitive stage
**Cognitively unimpaired (CU)**	-	-	-	Normal AD biomarkers, CU
	
	**+**	-	-	Preclinical Alzheimer’s pathologic change, CU
	
	**+**	**+**	-	Preclinical AD
		
	**+**	**+**	**+**	
	
	**+**	-	**+**	Alzheimer’s and concomitant non-Alzheimer’s pathologic change, CU
	
	-	**+**	-	Non-Alzheimer’s pathologic change, CU
		
	-	-	**+**	
		
	-	**+**	**+**	

**Mild cognitive impairment (MCI)**	-	-	-	Normal AD biomarkers, MCI
	
	**+**	-	-	Alzheimer’s pathologic change with MCI
	
	**+**	**+**	-	AD with MCI (prodromal AD)
		
	**+**	**+**	**+**	
	
	**+**	-	**+**	Alzheimer’s and concomitant non-Alzheimer’s pathologic change, MCI
	
	-	**+**	-	Non-Alzheimer’s pathologic change with MCI
	-	-	**+**	
	-	**+**	**+**	

**Dementia (DM)**	-	-	-	Normal AD biomarkers, MCI
	
	**+**	-	-	Alzheimer’s pathologic change with dementia
	
	**+**	**+**	-	AD with dementia
		
	**+**	**+**	**+**	
	
	**+**	-	**+**	Alzheimer’s and concomitant non-Alzheimer’s pathologic change, DM
	
	-	**+**	-	Non-Alzheimer’s pathologic change with dementia
		
	-	-	**+**	
		
	-	**+**	**+**	


*Only those with A biomarkers (A+) are considered to be on the AD pathological spectrum. Biomarkers for A (amyloid) include CSF Aβ_42_ and amyloid PET. Biomarkers for T (tau) include CSF p-tau and tau PET. Biomarkers for N (neurodegeneration) include CSF total tau, structural changes detectable by MRI (i.e., atrophy), and FDG-PET. Neuroimaging-detectable brainstem changes might inform the T or N categories, or potentially comprise a separate category in future iterations of the NIA-AA research framework.*

## Brainstem Changes in AD

Although considerable focus in the AD field has been placed on pathological changes in cortical, hippocampal, and basal forebrain regions, brainstem pathology has also been described since at least the 1930s (Hannah, 1936). The brainstem is a small and complex region, serving as a major relay center and signal integrator for the central nervous system (CNS). Rostro-caudally it is comprised of the midbrain, pons, and medulla, together containing the majority of neurons belonging to several widely projecting monoaminergic modulatory systems. These monoamine transmitters include serotonin (5-HT) and the catecholamines dopamine and noradrenaline (NA), the dysfunction of which have been well-described in AD (Trillo et al., 2013; Šimić et al., 2017). Early and severe AD-associated changes occur in the major brainstem serotonergic and noradrenergic nuclei, the dorsal raphe nucleus (DRN) and locus coeruleus (LC), respectively, a brief summary of which is provided below. For more in depth reading, several comprehensive reviews have been published (Szabadi, 2013; Trillo et al., 2013; Giorgi et al., 2017; Šimić et al., 2017).

### The Dorsal Raphe Nucleus

The DRN is one of a cluster of serotonergic nuclei, located near the midline in the dorsal midbrain and pons. It contains the majority of brainstem serotonergic neurons and is comprised of about 235,000 neurons in the adult human brain, roughly 70% of which produce 5-HT. The DRN sends ascending projections to regions such as the cortex, hippocampus, and striatum, as well as descending projections to the lower brainstem and spinal cord (Šimić et al., 2017). The serotonergic neurons of the DRN undergo substantial cell loss in AD (Lyness et al., 2003), and tangle pathology occurs early in the disease process (Rüb et al., 2000; Grinberg et al., 2009; Braak et al., 2011; Ehrenberg et al., 2017). Dysfunction in the serotonergic system has been strongly implicated in the behavioral and psychological symptoms of dementia (BPSD), such as agitation, aggression, hallucinations, and depression (Lanctôt et al., 2001). In particular, depressive symptoms later in life appear to be a feature of preclinical neurodegenerative changes, even before the onset of cognitive decline (Donovan et al., 2015; Singh-Manoux et al., 2017). Similarly, the DRN (along with the LC) is heavily involved in regulation of the sleep/wake cycle and cognitively normal individuals meeting criteria for preclinical AD have measurably worse sleep quality versus controls (Ju et al., 2013).

### The Locus Coeruleus

The LC is a long and narrow nucleus in the brainstem pons, averaging about 14.5 mm long and up to about 2.5 mm thick (Fernandes et al., 2012). It extends caudally along the cerebral aqueduct from the level of the inferior colliculus, ending ventrolateral to the floor of the fourth ventricle. It consists of only about 20 to 30,000 neurons per side in the healthy adult (German et al., 1988; Baker et al., 1989). Despite its small size, the LC provides the majority of central noradrenergic innervation via extensive projections throughout the CNS and it plays important roles across behavioral, cognitive, and physiological domains. It has also been implicated in early BPSD changes (Matthews et al., 2002) and tangle pathology in the LC occurs early and progressively throughout the disease process, even prior to tangle pathology within the DRN (Ehrenberg et al., 2017). Interestingly, the first detectable tau pathology within the brain is in the LC, appearing even in cognitively normal young individuals (Braak et al., 2011), and it has been speculated that LC damage and noradrenergic dysfunction might potentiate pathology in target regions such as cortex and hippocampus (Heneka et al., 2010; Braun et al., 2014; Feinstein et al., 2016). The LC undergoes severe topographic degeneration in AD, such that areas containing neurons reciprocally connected to hippocampal and cortical forebrain regions are selectively lost (Marcyniuk et al., 1986; Lyness et al., 2003; Zarow et al., 2003; Theofilas et al., 2017), and LC degeneration correlates with cognitive dysfunction (Grudzien et al., 2007). It is important to note here that the distinct but common condition known as primary age-related tauopathy (PART) also manifests with early LC tau pathology (Nelson et al., 2016). AD-related LC tau pathology by definition refers only to that co-occurring with amyloid biomarker changes (*e.g.*, reduced CSF Aβ_42_, increased cortical amyloid PET).

Although the DRN and LC are quite small, the pathology within them occurs early, progressively, and severely, increasing the likelihood of neuroimaging-based detection in the preclinical phase of disease. How this might practically be achieved is the topic of the following section.

## Neuroimaging the Brainstem

Of the non-invasive imaging modalities available, MRI and PET are those most commonly used in AD research (**Table 2**). MRI is primarily used to rule out other causes of dementia (e.g., stroke) and assess brain structural alterations informative of neurodegenerative change (Kehoe et al., 2014). It can also measure functional parameters relevant to AD pathology such as tissue perfusion (Wolk and Detre, 2012) or blood brain barrier (BBB) integrity (Raja et al., 2017); however, these methods have yet to be validated for biomarker use. PET in the AD context is employed primarily for assessment of levels of Aβ or tau, and to measure brain glucose hypometabolism with FDG-PET (Bao et al., 2017; Rice and Bisdas, 2017; Villemagne et al., 2018). These measurements are taken in relevant cortical and hippocampal regions, due in part to logistical considerations that have limited the use of these modalities for brainstem evaluation. The brainstem is a small structure with substantial anatomical complexity, requiring high resolution to visualize its component structures, and there is little endogenous contrast to enable the easy separation of nuclei. Additionally, significant physiological noise is present from cardiorespiratory systems, making fine-grained measurements difficult even with higher resolution systems (for review see Sclocco et al., 2017). Nonetheless, refined techniques are increasing the sensitivity of brainstem measurement, and a recent MRI study successfully identified reductions in dorsal midbrain volume in AD patients, corresponding to the location of the raphe nuclei (Lee et al., 2015). The increasing adoption of ultra-high-field (UHF) magnets for MRI (Deistung et al., 2013) and the High-Resolution Research Tomograph (HRRT) for PET (Schain et al., 2013), along with improved analytical methods (Iglesias et al., 2015), are enhancing the accessibility of these small brainstem regions to neuroimaging.

**Table 2:** Validated and potential neuroimaging-based biomarkers for AD-relevant pathology.

	Pathology	Imaging technique/sequence
**Validated AD neuroimaging biomarkers^1^**	Amyloid (A)	Amyloid PET
	Tau (T)	Tau PET
	Hypometabolism (N)	FDG-PET
	Atrophy (N)	Structural MRI
**Potential AD neuroimaging biomarkers**	Perfusion	Arterial spin labeling, contrast-enhanced MRI
	BBB integrity	Contrast-enhanced MRI
	Vascular reactivity	Blood-oxygen-level-dependent MRI
	Functional connectivity	Blood-oxygen-level-dependent MRI
	Neurotransmitter dysfunction	Neuromelanin-sensitive MRI, MRS, PET
	White matter disruption	Diffusion tensor imaging
	Neuroinflammation	MRS, translocator protein PET^∗^
	Synaptic dysfunction	Synaptic vesicle glycoprotein 2A PET^∗^


*Included are pathologies that have been reported in AD and techniques used to image them *in vivo*. The techniques listed have also been successfully applied to the brainstem in the published literature in various contexts, with the exception of the newer PET indicators of neuroinflammation and synaptic dysfunction, marked by ^∗^. MRS, magnetic resonance spectroscopy. ^*1*^Validated AD neuroimaging biomarkers are those described in the recently updated NIA-AA research framework (Jack et al., 2018).*

### Neuroimaging the DRN

The imaging of individual brainstem raphe nuclei poses some challenges (Kranz et al., 2012). However, it has primarily been achieved with the use of PET radioligands specific for 5-HT receptors or the 5-HT transporter (Kranz et al., 2012; Paterson et al., 2013; Schain et al., 2013; Kumar and Mann, 2014; Zhang et al., 2017). There are only two studies directly assessing the DRN *in vivo* in AD spectrum changes, both using functional MRI (fMRI). In one, reduced default mode network connectivity of the DRN was found in patients with AD, distinctive from changes observed in dementia with Lewy bodies (Zhou et al., 2010). A combined PET and fMRI study found reduced 5-HT transporter in the DRN of patients with MCI versus controls, associated with reduced functional connectivity between the hippocampus and DRN (Barrett et al., 2017). In addition, neuroimaging alterations in DRN are observable in patients with anxiety (Lanzenberger et al., 2007; Lee et al., 2011; Johnston et al., 2015; Pillai et al., 2018) and major depressive disorder (Lee et al., 2011; Johnston et al., 2015; Pillai et al., 2018), indicating that such changes might also be detectable in patients with BPSD due to AD. Interestingly, one group has successfully used a fusion PET/MRI system with both HRRT PET and UHF MRI (7T) for brainstem serotonergic imaging (Cho et al., 2007). With this system, all 5 individual raphe nuclei have been visualized using UHF MRI and FDG-PET or ^11^C-DASB PET (a radioligand for the 5-HT transporter) (Son et al., 2014). This raises the immediate possibility of testing the hypothesis that neurodegenerative biomarker changes may be observable in the DRN in early stages of AD. Whether such a system might also be able to detect other pathology (e.g., tau PET) in the DRN remains to be seen, but such a study would be well worth the effort. A major caveat is that the current cost and rarity of such systems limit their use; however, they are likely to become more common with time (Nensa et al., 2014), especially in light of demonstrated utility in dementia research (Dukart et al., 2011). Specific funding to equip Alzheimer’s Disease Research Centers with such systems would rapidly expand their use and facilitate research in this domain.

### Neuroimaging the LC

The biosynthesis of catecholamines in humans is associated with the production of neuromelanin, putatively a mechanism to reduce oxidative damage (Zecca et al., 2008). The noradrenergic LC is no exception, with neuromelanin levels increasing with age and then starting to decline around the seventh decade of life (Mann and Yates, 1974). Conveniently, neuromelanin has paramagnetic properties that allow regions with a high neuromelanin content to be directly imaged with T1-weighted or magnetization transfer weighted MRI scans on widely used 3T magnets (Sasaki et al., 2006, 2008). This neuromelanin-sensitive MRI (NM-MRI) allows for reliable mapping of the human LC (Keren et al., 2009, 2015; Tona et al., 2017). Although this property is increasingly being exploited, only two studies have applied this approach to AD patients *in vivo*. In one study, a non-significant reduction in neuromelanin signal versus controls was found, however, only 6 AD patients were included in the study (Miyoshi et al., 2013). A subsequent larger study of 22 AD, 38 MCI, and 26 controls reported significantly reduced LC neuromelanin signal in both MCI and AD patients as compared with controls, with no difference between MCI or AD groups (Takahashi et al., 2015). This latter finding indicates that neuronal loss might occur relatively early in these patients, even before clinically detectable cognitive impairment. Interestingly, a study of healthy individuals showed that the LC neuromelanin signal positively correlates with established proxies of neural reserve such as years of education (Clewett et al., 2016), a finding in line with other data that implicate the LC as a physiological substrate of cognitive reserve (Robertson, 2013; Wilson et al., 2013). Further, another recent study found that reduced LC neuromelanin signal was associated with poorer memory even in healthy older adults, particularly for emotionally negative events (Hämmerer et al., 2018). Such findings raise the possibility that LC neuromelanin signal may have prognostic value for older individuals in preclinical stages of AD, well before cognitive impairment.

In addition to NM-MRI, there have been studies examining LC functional connectivity in healthy and clinical populations. These studies have primarily focused on psychiatric symptoms, showing that LC functional connectivity with certain limbic regions is increased in patients with generalized anxiety disorder (Meeten et al., 2016), post-traumatic stress disorder (Steuwe et al., 2015), or those at high risk for schizophrenia (Anticevic et al., 2014). Whether such changes are also present in AD patients with BPSD has yet to be determined. Additionally, PET radioligands have been developed that bind the noradrenaline transporter (NET) (Stehouwer and Goodman, 2009; Adhikarla et al., 2016) and various adrenoceptor subtypes (Lehto et al., 2015; Phan et al., 2017). These have even been combined with NM-MRI, in a study demonstrating a correlation between LC neuromelanin signal and NET binding in certain LC projection areas in Parkinson’s disease (Sommerauer et al., 2018). Although various studies have found that NET density is decreased within the LC of the AD brain (Tejani-Butt et al., 1993; Szot et al., 2000; Gulyás et al., 2010), this has yet to be reported in AD patients *in vivo*.

## Summary

Thanks to recent advances in neuroimaging technologies, particularly UHF MRI, HRRT PET, and hybrid PET/MRI systems, the capacity to measure AD-associated brainstem pathology *in vivo* has reached a point where we can begin to assess its utility. Given the neuropathological findings described above, it is reasonable to expect alterations in these regions in at least a subset of patients with preclinical AD or even preclinical Alzheimer’s pathologic change (**Table 1**). One of the first questions to address is whether neuroimaging-detectable LC or DRN pathology, either alone or in some combination with other markers, can help predict which patients on the AD spectrum will go on to develop AD dementia. The LC in particular seems promising in light of the aforementioned evidence indicating its early loss in size (Theofilas et al., 2017) and its potential role as a substrate of cognitive reserve (Wilson et al., 2013; Clewett et al., 2016; Hämmerer et al., 2018). Additionally, the technology to assess its integrity *in vivo* is already in increasingly widespread use. Indeed, a recent systematic review of LC NM-MRI studies by Liu et al. (2017) identified areas where this technique can be improved, particularly in terms of standardization of LC signal measurements and methods of sub-regional analysis. This latter aspect may be especially useful given the topographic specificity of LC degeneration in AD. Additionally, the advent of new PET radioligands for synaptic dysfunction and neuroinflammatory change (Bao et al., 2017; **Table 2**) opens new possibilities. Neuroinflammation in particular is increasingly appreciated for its significant role in the early etiology of AD, and various MRS-detectable metabolites have historically been taken as indicators of neuroinflammatory changes (Quarantelli, 2015). More recently, progress has been made in PET imaging of translocator protein (TSPO) as a biomarker of activated microglia and astrocytes (Kreisl et al., 2018).

Despite the promise, there is much to be studied before such strategies might be employed in the clinic. The aforementioned studies have been small in both size and number, and they need to be expanded and replicated. A more fundamental question of whether brainstem neuroimaging is likely to provide benefit above and beyond other biomarkers remains and, while it is too early to say, we believe that the potential is worth investigating as technological advancements allow it. Finally, whether or not neuroimaging within the LC or DRN becomes useful clinically, the techniques outlined here afford an opportunity to advance our understanding not only of AD pathological progression, but other neurodegenerative diseases as well.

## Author Contributions

DB wrote the manuscript. All authors conceived, revised, and approved the final manuscript.

## Conflict of Interest Statement

The authors declare that the research was conducted in the absence of any commercial or financial relationships that could be construed as a potential conflict of interest.
